# Chemical Analysis of Cellular and Extracellular Carbohydrates of a Biofilm-Forming Strain *Pseudomonas aeruginosa* PA14

**DOI:** 10.1371/journal.pone.0014220

**Published:** 2010-12-03

**Authors:** Charlène Coulon, Evgeny Vinogradov, Alain Filloux, Irina Sadovskaya

**Affiliations:** 1 Université Lille Nord de France, Lille, France; 2 Université du Littoral-Côte d'Opale, LR2B, Bassin Napoléon, Boulogne sur Mer, France; 3 Institute for Biological Sciences, National Research Council of Canada, Ottawa, Canada; 4 Division of Cell and Molecular Biology, Faculty of Natural Science, Centre for Molecular Microbiology and Infection, Imperial College London, London, United Kingdom; 5 Université du Littoral-Côte d'Opale, UMT 08, Boulogne sur Mer, France; Charité-Universitätsmedizin Berlin, Germany

## Abstract

**Background:**

*Pseudomonas aeruginosa* is a Gram-negative bacterium and an opportunistic pathogen, which causes persisting life-threatening infections in cystic fibrosis (CF) patients. Biofilm mode of growth facilitates its survival in a variety of environments. Most *P. aeruginosa* isolates, including the non-mucoid laboratory strain PA14, are able to form a thick pellicle, which results in a surface-associated biofilm at the air-liquid (A–L) interface in standing liquid cultures. Exopolysaccharides (EPS) are considered as key components in the formation of this biofilm pellicle. In the non-mucoid *P. aeruginosa* strain PA14, the “scaffolding” polysaccharides of the biofilm matrix, and the molecules responsible for the structural integrity of rigid A–L biofilm have not been identified. Moreover, the role of LPS in this process is unclear, and the chemical structure of the LPS O-antigen of PA14 has not yet been elucidated.

**Principal Findings:**

In the present work we carried out a systematic analysis of cellular and extracellular (EC) carbohydrates of *P. aeruginosa* PA14. We also elucidated the chemical structure of the LPS O-antigen by chemical methods and 2-D NMR spectroscopy. Our results showed that it is composed of linear trisaccharide repeating units, identical to those described for *P. aeruginosa* Lanýi type O:2a,c (Lanýi-Bergman O-serogroup 10a, 10c; IATS serotype 19) and having the following structure: -4)-α-*L*-GalNAcA-(1–3)-α-*D*-QuiNAc-(1–3)- α-*L*-Rha-(1-. Furthermore, an EC O-antigen polysaccharide (EC O-PS) and the glycerol-phosphorylated cyclic β-(1,3)-glucans were identified in the culture supernatant of PA14, grown statically in minimal medium. Finally, the extracellular matrix of the thick biofilm formed at the A-L interface contained, in addition to eDNA, important quantities (at least ∼20% of dry weight) of LPS-like material.

**Conclusions:**

We characterized the chemical structure of the LPS O-antigen and showed that the O-antigen polysaccharide is an abundant extracellular carbohydrate of PA14. We present evidence that LPS-like material is found as a component of a biofilm matrix of *P. aeruginosa.*

## Introduction

*Pseudomonas aeruginosa* is a Gram-negative bacterium, which can be found in soil, water, skin flora and many other natural and artificial environments. It is a cause of life-threatening lung infections in individuals with cystic fibrosis (CF), which are impossible to eradicate due to the biofilm lifestyle, adopted by this microorganism [1], [2].

Biofilms are microbial communities that grow on a surface or at an interface, and are embedded in a thick extracellular (EC) matrix [3]. EC matrix of *P. aeruginosa* biofilms contains extracellular DNA [4], proteins and exopolysaccharides (EPS), which are considered as key components of *P. aeruginosa* biofilm matrix [5]. Several recent studies have indicated that the switch from planktonic growth to biofilm mode is under the control of a complex regulatory network resulting in up-regulation of polysaccharide biosynthetic genes [6]–[9].

Alginate, an anionic polymer composed of mannuronic and guluronic acids, was traditionally considered the major EC matrix polysaccharide of mucoid *P. aeruginosa* [10]. However, several reports suggested that non-mucoid *P. aeruginosa* strains, representing most clinical and environmental isolates, do not produce much alginate but are still capable of forming fully mature biofilms [11]. The analysis of the *P. aeruginosa* genomic sequence revealed at least four additional gene clusters which appeared to encode proteins involved in exopolysaccharide biosynthesis. The structure of *p*olysaccharide *s*ynthesis *l*ocus (*psl*)-dependent polysaccharide has been recently established [12]. The *pel* (*pel*licle) gene cluster was shown to be involved in the formation of a pellicle, a surface-associated biofilm on the air-liquid (A–L) interface in standing cultures [13], [14]. A comparison of carbohydrates produced by *P. aeruginosa* PA14 and its isogenic *pel* deletion mutant suggested that *pel* genes are responsible for the production if a glucose-rich matrix material [13]. In our recent study; we characterized a family of glycerophosphorylated cyclic β-(1,3)-glucans, which were the main glucose-containing polymers of the extracellular matrix of *P. aeruginosa* PA14 and were coded by the *ndvB*, and not by the *pel* locus [15]. The nature of the Pel polysaccharide thus remains largely unknown.

The non-mucoid laboratory strain *P. aeruginosa* PA14 is able to form thick, shear-forces resistant, biofilms at the A–L interface. These biofilms (or pellicles) are reported to have a rigid, paper-like consistency [13]. Molecules responsible for the structural integrity of such biofilms have not been identified to date, but they may be extracellular (EC) or covalently bound to the bacterial membrane. The rigid pellicles of PA14 could not be disintegrated by mild procedures [13]. Harsh treatments, which were proposed to disintegrate the pellicle, such as solubilization in alkali [13], inevitably affect the bacterial cells and liberates LPS from the outer membrane. Therefore, the effective localization of carbohydrate polymers liberated with such a treatment (cellular or EC) remains uncertain. In order to have a better idea on the carbohydrate distribution, we first characterized the chemical nature of LPS, a major carbohydrate of the *P. aeruginos*a cell-wall. Indeed, the LPS structure of the common laboratory PA14 strain is still unknown. Subsequently, in an effort to identify the “scaffolding” polysaccharides component of *P. aeruginosa* PA14 biofilms, we performed a systematic analysis of the carbohydrate composition of the A–L biofilms of *P. aeruginosa* PA14. Knowledge of the nature of the EC polymers of PA14, which can potentially accumulate in the biofilm, could facilitate the task of establishing a correlation between putative genes encoding for key EPS biosynthesis and their products.

## Results

### Preparation of LPS and structural analysis of the LPS O-antigen

We extracted LPS of PA14 cells by standard hot phenol-water method [16] and characterized this molecule by monosaccharide and fatty acids composition analysis. Monosaccharide analysis of the LPS showed the presence of rhamnose (Rha), 2-diacetamido-2,6-dideoxy-glucose (*N*-acetyl-quinovosamine, Qui*N*Ac), and glucose (Glc); as well as small amounts of *N*-acetyl-glucosamine (Glc*N*Ac) and heptose (Hep), characteristic for the lipid A-core region of LPS. Total fatty acid analysis showed the following main components: 2- and 3-hydroxydodecanoic acid (2-OH C_12∶0_ and 3-OH C_12∶0_; ∼20%); n-hexadecanoic acid (C _16∶0_; ∼32%), and n-octadecanoic acid (C_18∶0_; 31%). Smaller amount of unsaturated octadecenoic acid (C_18∶1_; ∼9%) and fatty alcohols (hexadecanoic alcohol, C 16-ol, 3%, and octadecanoic alcohol, C:18-ol, 9%) were also present.

**Figure 1:**
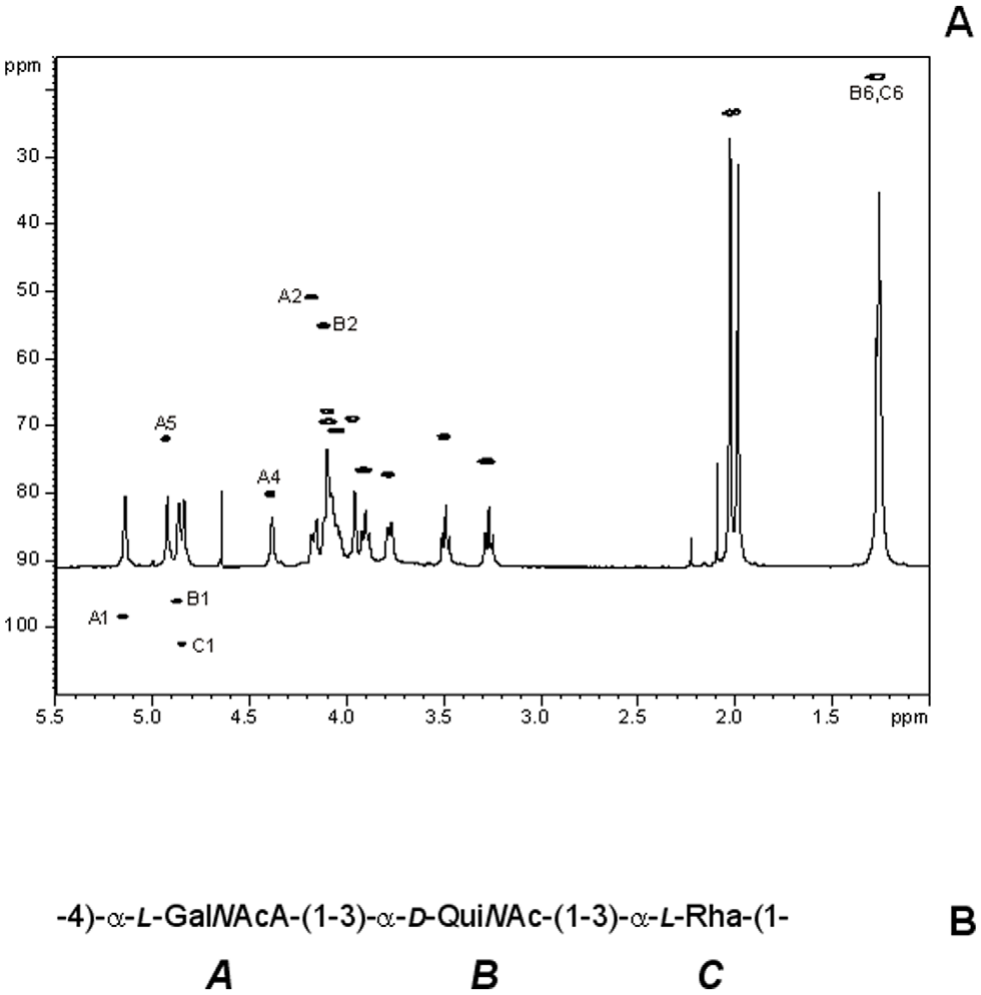
NMR spectra and the structure of the O-PS. ^1^H-^13^C HSQC spectrum along with the corresponding 1D ^1^H-NMR spectrum (A), and the structure of the trisaccharide repeating unit (B) of the LPS O-antigen (O-PS) from *P. aeruginosa* PA14.

LPS was hydrolyzed by acetic acid and the O-antigen polysaccharide (O-PS) purified on a Sephadex G-50 column. The detailed chemical structure of the O-PS was elucidated by NMR spectroscopy. The HSQC spectrum (Fig. 1, A) indicated the presence of three anomeric signals A1, B1, and C1; at least two nitrogen-bearing carbons (A2 and B2), and two *N*-acetyl groups. This suggested that the polymer was composed of trisaccharide repeating units, with two sugar residues corresponding to 2-deoxy-2-aminosugars. ^1^H and ^13^C spectra of the O-PS were fully assigned using 2D homo- and heteronuclear correlation techniques. Spectra contained signals of a spin system of 2-acetamido-2-deoxy-α-L-galacturonic acid (Gal*N*AcA, unit A), substituted at position 4; and spin systems of 3-substitued 2-acetamido-2,6-dideoxy-α-D-glucose (Qui*N*Ac, unit B) and α-L-rhamnose (Rha, unit C). The sequence of the repeating unit (A–B–C) was established by inter-residue NOEs.

The assignments are summarized in Table 1, and the corresponding structure is schematically shown in Fig. 1, B. This structure was identical to the one described for *P. aeruginosa* type O:2a,c Lanýi classification (O-serogroup 10a,10c of Lanýi-Bergan or serotype 19 of IATS classification [17],[18]). The ^13^C assignments closely corresponded to the structure described previously [17].

**Table 1:** ^1^H and ^13^C chemical shifts of *P. aeruginosa* PA14 O-PS.

Unit	H/C 1	H/C 2	H/C 3	H/C 4	H/C 5	H/C 6a	H/C 6b
α-GalNAcA A	5.16	4.17	4.10	4.40	4.97		
	*98.2*	*50.7*	*67.6*	*80.0*	*71.6*		
α-QuiNAc B	4.87	4.12	3.90	3.28	4.10	1.26	
	*95.9*	*54.9*	*76.4*	*75.2*	*69.2*	*17.8*	
α-Rha C	4.84	3.95	3.78	3.50	4.05	1.26	
	*102.3*	*68.8*	*77.1*	*71.4*	*70.5*	*17.8*	

Spectra were recorded at 25°C in D_2_O with acetone standard. ^13^C chemical shifts are shown in italic.

The O-antigen is composed of the trisaccharide repeating units, containing 2-acetamido-2-deoxy-α-L-galacturonic acid (α-L-Gal*N*AcA, unit A), 2-diacetamido-2,6-dideoxy-α- D-glucose (α-D-Qui*N*Ac, unit B); and α-L-rhamnose (α-L-Rha, unit C) (Fig. 1, B). Typically for *P. aeruginosa* species [18], the PA14 LPS O-antigen is thus an acidic polysaccharide, rich in aminosugars.

### Identification of extracellular carbohydrates produced in chemically defined medium

**Figure 2:**
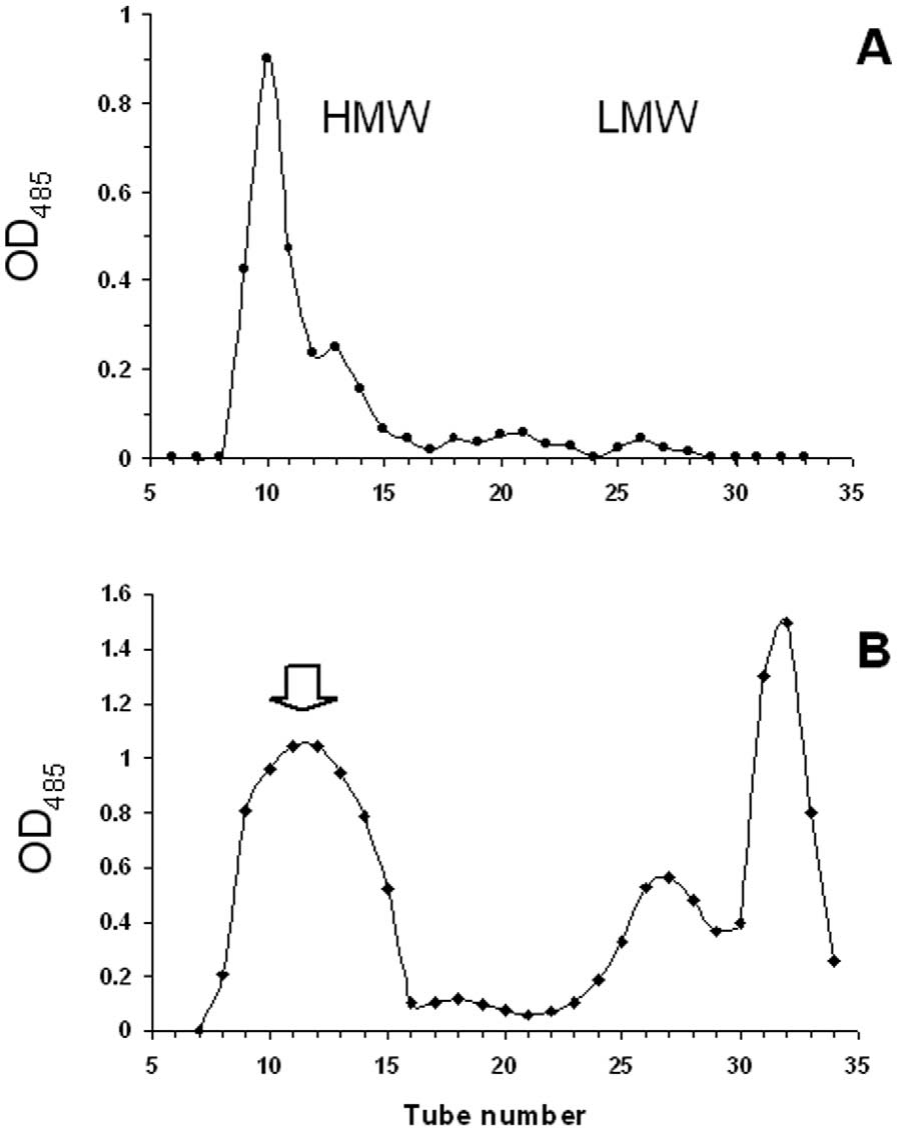
Typical elution profiles of crude carbohydrate extracts of the EC biofilm matrix (A) and culture medium (B) of *P. aeruginosa* PA14 on Sephadex G-50 column. Aliquots (400 ml) of each 5-ml fraction were assayed colorimetrically for neutral sugars [52]. Fractions used for NMR analysis are marked with an arrow.

We further carried out a systematic study of polysaccharides, produced by PA14 grown in standing cultures and in chemically defined M63 medium. Knowing that *P. aeruginosa* cells often release large quantities of extracellular carbohydrates in the growth medium [12], we examined the carbohydrate extracts both from the cell-associated EC matrix and from the growth medium. The extracts were prepared using the procedure developed earlier, including the precipitation of the EC DNA (eDNA) and proteins by TCA, followed by complete deproteination by phenol-chloroform extractions [12], [15]. The crude carbohydrate preparations were fractionated on the Sephadex G-50 column, and corresponding fractions screened by ^1^H-NMR and composition analysis (Figs. S1 and 2). Typically, most of carbohydrate material of the cell-associated EC matrix was eluted in the void volume of the column (Fig. 2, A), and contained Rha, Qui*N*Ac and Glc in different ratios. In addition, small amounts of Glc*N*Ac and Hep were identified, indicating the presence of LPS. ^1^H-NMR showed the presence of the β-(1,3)-glucans [15] and the O-PS (described below) as main components of the high molecular weight (HMW) fraction.

**Figure 3:**
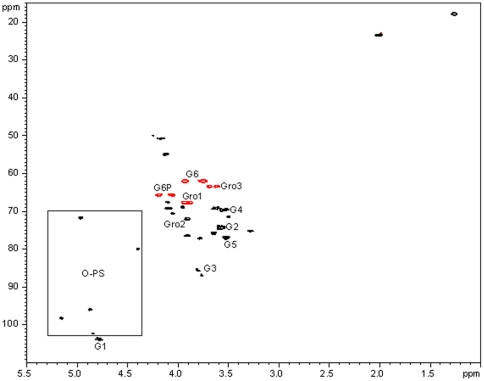
^1^H-^13^C HSQC spectrum of the EC carbohydrate of the growth medium of *P. aeruginosa* PA14. Signals corresponding to the β-(1,3)-cyclic glucans are labelled; G =  Glc, Gro  =  glycerol. Signals in square and other unlabeled signals belong to the EC O-PS.

Carbohydrate material of the growth medium was eluted as a large peak close to the void volume (Fig. 2, B). It was collected, lyophilized, and solubilized in deuterium oxide. LPS was removed by high-speed centrifugation (see Materials and Methods), and the clear supernatant analyzed by 1- and 2-D NMR techniques. NMR analysis revealed the presence of two components (Fig. 3): a polysaccharide composed of the trisaccharide repeating units identical to one of the O-PS (Table 1), and the cyclic glycerophosphorylated β-(1,3)-glucan.

These results allowed us to conclude that LPS, the EC O-PS, and a family of cyclic glycerophosphorylated β-(1,3)-glucans are three main extracellular carbohydrates released by *P. aeruginosa* PA14 in M63 medium.

### Chemical analysis of the A-L biofilms

**Figure 4:**
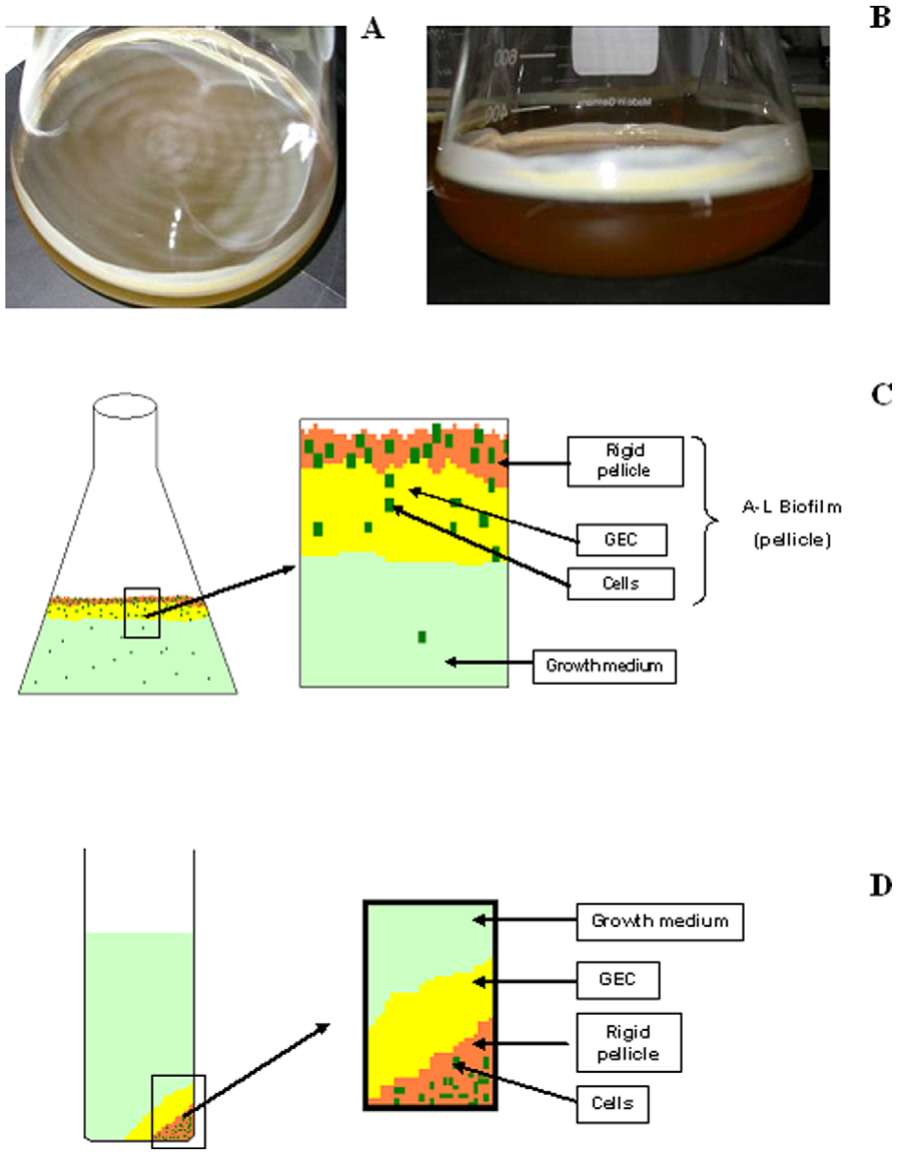
A-L biofilms of *P. aeruginosa* PA14 grown in standing cultures. Cells were grown in T-medium in 2-l Erlenmeyer flask at 25°C for 6 days. Photographs: top (A) and side view (B); schematic representation of the culture in the flask (C) and after centrifugation (D). Cells are shown in dark green, growth medium – in light green, rigid pellicle in orange and gelatinous EC matrix (GEC) in yellow.

Growth in standing cultures in T-broth in Erlenmeyer flasks at room temperature are the conditions known as most favorable for the A-L biofilm formation of PA14 [13],[19]. When grown under these conditions, *P. aeruginosa* PA14 forms a rigid pellicle with a loosely adherent thick layer of a viscous transparent gelatinous EC matrix (GEC) (Fig. 4A, B and C). Upon centrifugation of the cell culture, bacterial cells (and supposedly a part of the “rigid pellicle” holding the cells together) settle to the bottom of the centrifuge tube, while the GEC forms an adherent layer between the cells and the media (Fig. 4, D). The GEC matrix could be detached from the bacterial cells by mild procedures, such as mild sonication or shaking with 5-mm diameter glass beads.

Knowledge of the chemical structure of main carbohydrate polymers of PA14 enabled us to make the preliminary analysis of its A–L biofilm using isolation procedures which would minimize the loss of carbohydrate material (Fig. S2).

The soluble GEC detached by glass beads was dialysed, lyophilized and used for preliminary analysis (Fig. S2). Cells and the “rigid pellicle” were solubilized in 1 M NaOH, as recommended by Friedman and Kolter [13].Carbohydrates were prepared as described in Materials and Methods and shown in Fig. S2, and subjected to dephosphorylation followed by composition methylation analysis. In agreement with the previous findings [13], methylation analysis revealed the presence of 3-linked Rha and 3-linked 6-deoxyhexosamine residues, which is in accordance with the proposed structure of the LPS O-antigen (Fig. 1, B). Smaller amounts of terminal Glc and 3-linked Hep could be accounted for the LPS core oligosaccharide. The 2-linked Rha was not detected, which indicated the absence in the pellicle of the A-band LPS O-antigen [20]. The 3-linked Glc most probably corresponded to the cyclic glycerophosphorylated β-(1,3)-glucans [15]. Minor amounts of 2-linked Glc pointed out to the possible presence of the short branched β-(1,2)-glucan [21], [15]. Overall, data of chemical analysis of the preparation were consistent with the presence of the LPS and β-(1,3)-glucans as the main carbohydrates. Our analytical methods could not allow us to identify any carbohydrates specific for the formation of the rigid pellicle.

The chemical composition of the GEC preparations was studied by colorimetric assays, DOC-PAGE, agarose gels, and monosaccharide composition analysis. Agarose gel profiles of the GEC preparations were identical to chromosomal DNA of this strain (Fig. S3). The amount of DNA could be estimated as up to 30% of the dry weight of the crude EC preparation. This is consistent with previous studies that demonstrate that DNA was the main component of EC matrix of several *P. aeruginosa* strains, in particular PAO1 [4], [22], [23].

**Figure 5:**
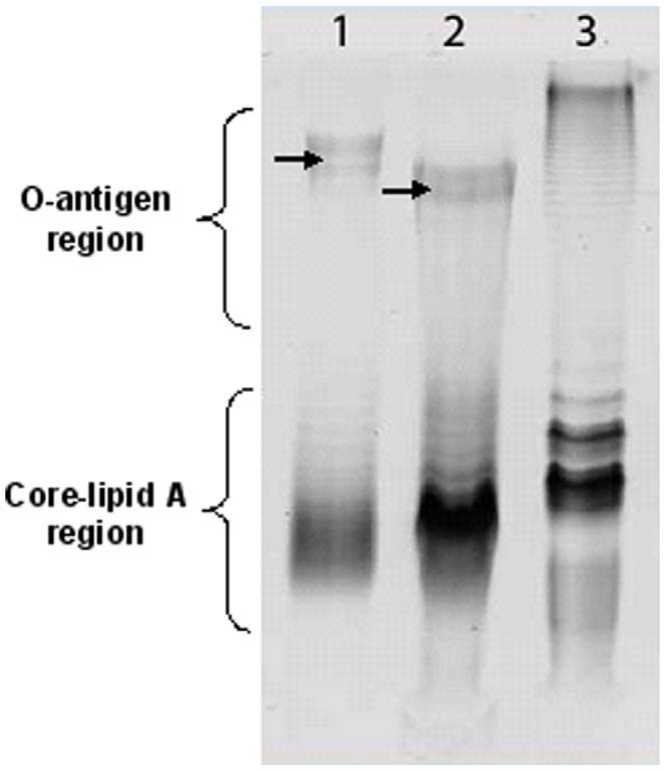
DOC-PAGE analysis of crude GEC extract of from *P. aeruginosa* PA14 A–L biofilm. Lane 1, crude GEC extract of from *P. aeruginosa* PA14 pellicle (30 µg); lane 2, *P. aeruginosa* PA14 LPS control (15 µg); lane 3, *Salmonella enteriditis* LPS control. O-antigen and core-lipid A regions of the LPS are indicated. Corresponding HMW bands of the LPS and GEC are indicated with arrows.

Furthermore, colorimetric assays and DOC-PAGE indicated that at least 20–25% of dry weight of this material consisted of LPS or LPS-like product. Interestingly, the DOC-PAGE profile of the GEC preparation was similar, but not identical, to the one of LPS extracted from cells by hot phenol-water method: In GEC, bands corresponding to the core-lipid A region were of relatively lower intensity, and O-antigen pattern indicated higher MW bands (Fig. 5). Main monosaccharide components of the GEC preparations were Rha and Glc along GlcN and Hep, characteristic for the core-lipid A region of the LPS. GEC produced it T-medium also contained Man and ribose (Rib). Unlike the PA14 LPS, only small amounts or no QuiNAc was detected, depending on the preparation. The combined data indicate that GEC contains, along with eDNA, Kdo-containing LPS-like material, which may be different from the cell-wall LPS of *P. aeruginosa* PA14. The detailed identification of this material requires further elucidation, and is currently under investigation.

### Comparative studies of extracellular carbohydrates of the wild type and pel mutants

EC and cell-associated carbohydrate extracts were prepared for the PA14Δ*pelC* mutant using the same procedures as for the wild-type (WT) strain. Monosaccharide composition, fatty acid and methylation analysis of corresponding fractions did not show significant differences between the two strains. Consistent with our previous studies [15], the glycerophosphorylated cyclic β-(1,3)-glucans were present in EC matrix of the PA14Δ*pelC* strain. eDNA was also detected in the GEC of the mutant strain (Fig. S3, lane 5) and the DOC-PAGE profile of the LPS-like material of GEC was similar to one of the WT strain (data not shown). In an attempt to answer the question if the production of the EC O-PS was related to the function of the *pel* locus, we prepared the EC and cell-associated carbohydrate extracts of the WT and Δ*pelC* mutant strains, as described in Materials and Methods. Crude extracts were fractionated on a Sephadex G-50 column, and the corresponding HMW fractions were analysed by ^1^H-NMR spectroscopy and DOC-PAGE. ^1^H-NMR spectra characteristic for O-PS were detected in the WT, but not in the corresponding Δ*pelC* preparations. These data thus suggest that *pel* locus could be involved in the production of the EC O-PS.

**Figure 6:**
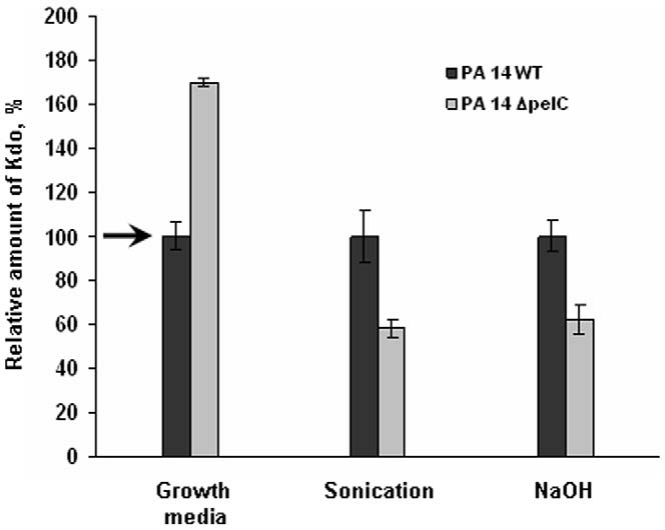
Comparative analysis of Kdo-containing material released in the growth medium and associated with cells for *P. aeruginosa* PA14 (WT) and its isogenic Δ*pelC* mutant. Cells were grown in M63 medium (3 ml of inoculum at OD of 0.0025 in 12-ml plastic tubes) at 25°C statically for 6 days. Extracts were prepared by sonication of the cell pellet in saline or by solubilizing it in 1 M NaOH (see Materials and Methods). Kdo-assays for the WT preparations are set at 100% and shown with an arrow. Data represent a typical experiment performed in triplicate. Experiments were performed at least three times by two researchers independently.

We also attempted to estimate the correlation between the phenotypic difference of the WT and Δ*pelC* strain and the amounts of carbohydrate material produced by the cells and accumulated in the biofilm matrix. We compared the quantities of EC LPS-like component of two strains, grown in chemically defined M63 medium at 25°C, optimal temperature for the expression of the *pel* genes [19]. Crude extracts were prepared as described in Materials and Methods, and quantities of Kdo-containing carbohydrate material were compared. Overall, we observed an accumulation of the cell-associated material and a decreased amount of the material released in the growth media, in the WT strain compared to the isogenic Δ*pelC* mutant (Fig. 6). This may indicate that *pel* genes are involved in the production of a factor(s) which favour an accumulation the EC carbohydrates, and in particular the Kdo-containing polymers, within the complex network of the A-L biofilm.

## Discussion

Nonmucoid strains of *P. aeruginosa* are the predominant environmental phenotype, and are also involved in colonization of CF lung at early stages of infection. It was shown that alginate is not a major component of the EC matrix of two nonmucoid laboratory strains, PAO1 and PA14 [11], and EC biofilm matrix of PAO1 consisted primarily of DNA [4], [22].

*P. aeruginosa* PA14 is a strain that has traditionally been used to study biofilms. This is largely due to its ability to form strong biofilm at the A–L interface. In the present work, we attempted to identify the carbohydrate polymers of the PA14 EC matrix. Knowledge of the nature of carbohydrate polymers which constitute the A–L biofilm of this strain could significantly facilitate establishing the identity of genes involved in their biosynthesis and, consequently, in the biofilm formation process.

LPS is the major carbohydrate component of the cell envelope of Gram-negative bacteria, and has been proposed to be involved in bacterial attachment to abiotic surfaces and biofilm formation [24], [25]. We showed that PA14 LPS O-antigen was composed of a trisaccharide repeating unit and contained Rha, QuiNAc and a 2-acetamido-2-deoxy-glacturonic acid (Fig. 1). Typically for *P. aeruginosa* LPS, PA14 O-PS is an anionic polymer, rich in amino-sugars and containing 6-deoxy-sugar residues [18]. Analysis of the extracellular polysaccharides released in the growth medium allowed us to identify a family of highly glycero-phosphorylated cyclic β-(1,3)-glucans [26], [15] and an acidic extracellular polysaccharide of *P*. *aeruginosa* PA14, which is composed of trisaccharide repeating units with a structure identical to the LPS O-antigen.

Goldman and co-workers first showed that *E. coli* serotype O11 expressed a half of its O-antigen in the LPS-unlinked capsular form [27]. Such “O-antigen capsules”, with the structure of repeating unit identical to the LPS O-antigen but not linked to the lipid A-core were later described for a number of gram-negative bacteria (reviewed in [28]). “O-antigen capsules” of *E. coli* were assigned to Group 4 capsules [29]. *Salmonella enteriditis* [28] and *Francisella tularensis* [30] were shown to produce capsular polysaccharides (CPS) structurally identical to the LPS O-antigen, but expressed by distinct genetic loci and relying on a separate biosynthetic and transport apparatus. Presence in the CPS preparations of *S. enteriditis* of octadecanoic acid and unsaturated fatty acids, which are not normally seen in LPS of Gram negative bacteria and are rather common for phospholipids of bacterial membranes, led the authors to speculate that the lipid anchor for this CPS could be a phospholipid(s).

To date, the presence of CPS has not been described for *P. aeruginosa*. However, a HMW immunogenic, non-toxic form of the LPS O-antigen has been previously isolated from culture supernatants of several *P. aeruginosa* strains [31]–[34] after prolonged growth. Isolation procedure of these PS included acid treatment, and therefore no conclusion about the presence of a possible lipid anchor could be drawn. Similarly to the case of *Salmonella* CPS [28], occurrence of hexadecanoic, octadecanoic and unsaturated fatty acids in our LPS and EC preparations could point out to a possible phospholipids anchor for the EC O-PS in *P. aeruginosa* PA14.

Knowledge of the chemical identity of the cell-wall LPS and EC carbohydrates allowed us to make a preliminary analysis of the composition of A–L biofilms of PA14. These biofilms formed in T-broth comprise rigid shear-forces resistant pellicle containing bacterial cells, and a loosely adherent transparent gelatinous material (Fig. 4). In *P. aeruginosa* PAO1, similar gelatinous material of solid surface-associated (SSA) biofilms was reported to consist mainly of DNA [22]. Our results showed that the EC material of A-L and SSA biofilms of PA14 contained, in addition to eDNA, up to and 25-30% (dry weight) of Kdo-containing LPS-like material. This is the first evidence of accumulation of an extracellular LPS-like material as an important “scaffolding” component of *P. aeruginosa* biofilms.

Accumulation of LPS in the A-L biofilm is consistent with the physico-chemical properties expected for a molecular framework build up at the interface between air and liquid. Formation of an A-L biofilm would require the presence of components with both hydrophilic and hydrophobic properties. The amphiphilic LPS molecule with an anionic, viscous and highly hydrated O-PS, seems to be a good candidate for this function.

Previous studies pointed out LPS as a candidate molecule that could provide structural integrity of *P. aeruginosa* biofilms [11]. *P. aeruginosa* LPS was shown to contribute to biofilm function and architecture by influencing bacterial adhesion, cell-to-cell adherence, and viscoelastic properties of biofilms [35]. Abraham *et al.* [36] recently demonstrated that *P. aeruginosa* LPS is able to form stable monolayer at the air-water interface. It is therefore tempting to speculate that formation of such monolayer by the amphiphilic LPS molecules may occur in initial steps of pellicle formation. Otherwise, uppermost layers of the pellicle might be formed by cells with increased cell surface hydrophobicity. Interestingly, it is known that low concentrations of *P. aeruginosa* rhamnolipids cause a release of LPS from the outer membrane and an increase of the cell surface hydrophobicity [37]. The product of *algC*, one of the alginate biosynthetic genes, whose expression is induced upon attachment to a surface [38], is also involved in the biosynthesis of rhamnolipid and LPS [11]. These phenomena may play an important role in the mechanisms of formation of biofilm and in particular A-L biofilms in *P. aeruginosa.*

The spontaneous release of LPS by Gram-negative bacteria during normal growth is a well-established phenomenon, which gave rise to the term “free endotoxin” [39], [40]. It was shown that *P. aeruginosa* [40], *Neissseria* [41], *Vibrio cholerae* [42], *E. coli* and *Salmonella typhimurium* [43] release the “free LPS” by a process apparently distinct from cellular autolysis. The mechanism of this release is not well established. One known mechanism of LPS release in *P. aeruginosa* during normal growth occurs *via* the production of membrane vesicles [44].

To our knowledge, this and our previous study [15] are first examples of a cyclic β-glucans as components of bacterial biofilm matrix. However, other glucose polymers were reported to play an important role in the formation of a biofilm at the A-L interface. For example, overproduction of cellulose and its acetylated form was causing the colonization of the A-L niche by *Pseudomonas fluorescens* and *Salmonella* spp. [45], [46], [47]. Biofilm formation in *Salmonella* is associated with the multicellular behaviour, which is characterized by the elaboration of thin aggregative fimbriae (Tafi), cellulose, and a yet uncharacterized EPS [48]. It is of interest that O-Ag capsule of *S. enteriditis* was found to play an essential role in the protection of cells against desiccation stress *via* the formation of a hydrated gel, but did not affect the formation of the extracellular network between cells [49]. In case of a wrinkly spreader of *P*. *fluorescens*, it was shown that cellulose fibers and LPS were required for strength and structural integrity of A-L biofilms, and defects in LPS expression affected the A-L biofilm strength [45]. Friedman and Kolter [13] have demonstrated that cellulose was not produced by *P. aeruginosa*. It is possible to hypothesise that cyclic β-(1,3)-glucans, which are known as good molecular chelating agents and are capable to bind to aminoglycoside antibiotic [26], [15], are also able to form molecular complexes with other components of the pellicle matrix, such as proteins, lipids, polysaccharides or LPS within of *P. aeruginosa* biofilms.

In conclusion, in this work we identified three abundant extracellular carbohydrates of *P. aeruginosa* PA14: LPS-like material, EC O-PS and the cyclic β-(1,3)-glucan and provide the experimental evidence of accumulation of extracellular LPS-like material and cyclic β-(1,3)-glucans in the *P. aeruginosa* biofilm matrix. Understanding the involvement of these carbohydrates in the biofilm formation process of different non-mucoid *P. aeruginosa* strains is an attractive challenge and yet requires further investigation.

## Materials and Methods

### Bacterial strain and growth conditions

*P. aeruginosa* PA14 was used for all studies. Cells were grown in Erlenmeyer flasks (500 ml per 2-liter flask, 75 ml per 250-ml flask) at 37°C for 3 days in M63 minimal medium; supplemented with 0.5% Casaminoacids ((Difco), 1 mM MgCl_2_, and 0.4% Glc [50], or at 25°C for 6–7 days in T-broth medium (10 g l^−1^ bacto peptone, 5 g l^−1^ NaCl). Standing cultures were inoculated with plate-grown bacteria to an OD_600_ = 0.0025, as recommended by Friedman and Kolter [13]. For comparative studies, PA14Δ*pelC* mutant [14] was used. Standing cultures containing 3 ml of M63 medium were grown for 6 days at 25°C in 12-ml polystyrene culture tubes (17×100 mm, Greiner Bio-one). For LPS preparation, cells were grown in Erlenmeyer flasks at 37°C for 24 hrs in LB-broth with shaking.

### LPS extraction and preparation of the LPS O-antigen

Cells were collected by centrifugation, washed with saline and extracted with 50% aqueous phenol at 65–70°C with intensive stirring [16]. The mixture was transferred into centrifuge tubes, cooled in ice, and the phases were separated by centrifugation (1 200 *g*, 4°C, 30 min). Joined aqueous and phenol phases were dialysed and lyophilised. The LPS preparation was further de-proteinated by TCA precipitation (5%), followed by dialysis and lyophilisation. This LPS preparation was used for a calibration curve in Kdo assays and as a control for DOC-PAGE.

For the preparation of the O-antigen, LPS was hydrolysed with 3% aqueous AcOH with stirring. Lipid A was removed by centrifugation, the supernatant lyophilised and fractionated on a Sephadex G-50 column. HMW fractions were collected and used for NMR analysis.

### Preparation of the carbohydrate extracts

The crude carbohydrate extract of the growth media and the extracellular biofilm matrix were prepared from M63 cultures and fractionated on Sephadex G-50 as described earlier [12]. The corresponding fractions were pooled and lyophilized. For NMR analysis, preparations were taken in 300 µl of D_2_O and centrifuged (14 000 *g*, 10 min); clear supernatants were used for NMR analysis.

For the chemical analysis of the pellicle, cell pellets which remained after mild sonication (rigid pellicle) were solubilized in 1 M NaOH at room temperature, with stirring for 10 min. The viscous solution was neutralized with glacial acetic acid, and the insoluble material removed by centrifugation (9 000 *g*, 15 min). Clear supernatant was diluted with water, dialyzed and lyophilized. It was used for monosaccharide composition and methylation analysis, preceded by dephosphorylation (51% hydrofluoric acid, 48 h, 4°C). Alternatively to alkaline treatment, the pellicles were extracted with hot phenol-water as described above.

### Preparation and analysis of the crude biofilm extract

Biofilm pellicles, formed in T-broth, were collected and washed gently with water. In order to detach the gelatinous extracellular material, they were suspended in water, and 5-mm glass beads were added to the suspension. The mixture was vortexed for 1 min, the cell suspension centrifuged (9 000 *g*, 20 min, 4°C). The remaining pellet was re-suspended in water and the procedure repeated. The clear supernatant represented the crude EC biofilm matrix solution and was used to assay the relative amount of LPS and DNA. Aliquots of this extract were used lyophilized to estimate the dry yield. The lyophilized material was further analyzed by agarose gel electrophoresis, DOC-PAGE, monosaccharide composition, fatty acid and methylation analysis. The remaining pellet (rigid pellicle) was solubilized in 1 M NaOH and analyzed as described above.

### DNA preparation

The eDNA from the growth media and GEC preparations was precipitated by 3 volumes of ethanol, collected by centrifugation and re-solubilized in water. Chromosomal DNA extractions were carried out on planktonic cultures of *P. aeruginosa* PA14 and the isogenic Δ*pel*C mutant, using Wizard® Genomic DNA purification kit according to the manufacturer's recommendations. DNA was subjected to electrophoresis on 1% agarose gels and visualized with GelRed™ (Interchim).

### Quantification of carbohydrates in the wild type and ΔpelC mutant strains

3-ml cultures were transferred in the eppendorf tubes, and cell with cell-associated material was collected by centrifugation. Cell pellets were washed gently with saline (2 ml). Joined supernatants (“Growth media”) were dialyzed, lyophilized, and suspended in water (2 ml). Cell pellets were suspended in saline (2 ml) and subjected to sonication (IKA Labotechnik sonicator, 50% intensity, 0.5 cycle, 3×10 sec) to give a “sonication” extract. In a separate experiment, the pellet was solubilized in 1 M NaOH (3 ml, “NaOH” preparation). In the three preparations, the amount of Kdo-containing material was assessed by the Kdo assay [51], using a purified PA14 LPS as a standard. Quantities were expressed in % of the PA14Δ*pelC* mutant compared to the WT strain.

### General and analytical methods

Gel-permeation chromatography was carried out on a Sephadex G-50 column (1.6×95 cm; Pharmacia), irrigated with water. Aliquots (200 µL) of each 5 mL fraction were assayed by colorimetry for aldose [52]. Carbohydrate samples were dephosphorylated by treatment with 48% HF (Acros Organic) for 48 hrs at 4°C.

Methylation analysis and GC were performed as described before [15]. Total fatty acids were liberated by hydrolysis with 4 M KOH (100°C, 16 h), and extracted by chloroform-methanol (2∶1) and hexane from neutralized solution. They were converted into methyl esters by methanolysis (2 M methanolic hydrogen chloride, 80°C, 4 h), acetylated by conventional methods.

GC-MS was performed with a Hewlett Packard mass spectrometer 5989A equipped with a fused silica capillary column, as described before [53].^ 1^H and ^13^C NMR spectra were recorded using a Varian Inova 500 MHz and 400 MHz spectrometers as described before [12].

Relative amount of LPS in crude samples was estimated using the Kdo assay [51], with a purified PA14 LPS as a standard. DOC-PAGE was performed as described by Reuhs et al. [54].

## Supporting Information

Figure S1 Schematic representation of the protocol used for purification of EC carbohydrates of *P. aeruginosa* PA14, grown in standing culture in M63 medium. Fractions obtained from cells, GEC and growth medium are color coded as explained in the legend of Fig. 4.
(2.36 MB EPS)

Figure S2 Schematic representation of the experimental protocol used for preliminary analysis of A-L biofilms of *P. aeruginosa* PA14, grown in standing culture in T-medium. T-medium is rich in complex carbohydrates and therefore was not analyzed. Fractions are color coded as explained in the legend of Fig. 4.
(1.87 MB EPS)

Figure S3 Agarose gel-electrophoresis of eDNA of crude GEC extract of from *P. aeruginosa* PA14 A-L biofilm and purified chromosomal DNA. Lane 1, Ladder 100 pb; Lane 2, purified chromosomal DNA of *P. aeruginosa* PA14 WT, lane 3, purified chromosomal DNA of *P. aeruginosa* PA14Δ*pel*C; lane 4, eDNA of crude GEC extract of from *P. aeruginosa* PA14 WT; lane 5, eDNA of crude GEC extract of from *P. aeruginosa* PA14Δ*pel*C.
(0.35 MB EPS)
